# Phytotoxic Strains of *Fusarium commune* Isolated from Truffles

**DOI:** 10.3390/jof10070463

**Published:** 2024-06-29

**Authors:** Anton Zvonarev, Vasily Terentyev, Valentina Zhelifonova, Tatiana Antipova, Boris Baskunov, Aleksander Avtukh, Tatiana Abashina, Aleksey Kachalkin, Mikhail Vainshtein, Anna Kudryavtseva

**Affiliations:** 1 G.K. Skryabin Institute of Biochemistry and Physiology of Microorganisms, Federal Research Center “Pushchino Scientific Center for Biological Research of the Russian Academy of Sciences”, Pushchino 142290, Russia; zvonarevibpm@gmail.com (A.Z.); avtukh@rambler.ru (A.A.); kachalkin_a@mail.ru (A.K.); 2 Institute of Basic Biological Problems, Federal Research Center “Pushchino Scientific Center for Biological Research of the Russian Academy of Sciences”, Pushchino 142290, Russia; 3 All-Russian Institute of Plant Protection, Pushkin 196608, Russia; 4 The Faculty of Soil Science, M.V. Lomonosov Moscow State University, Moscow 119234, Russia; 5 Independent Researcher, Moscow 115533, Russia

**Keywords:** *Fusarium*, mycelium, pigments, phytotoxicity, fatty acids, *Brassica juncea*, photosystem II

## Abstract

Most *Fusarium* species are known as endophytes and/or phytopathogens of higher plants and have a worldwide distribution. Recently, information discovered with molecular tools has been also published about the presence of these fungi in the microbiome of truffle fruiting bodies. In the present work, we isolated and identified three *Fusarium* strains from truffle fruiting bodies. All isolates were assigned to the same species, *F. commune*, and the strains were deposited in the All-Russian Collection of Microorganisms under accession numbers VKM F-5020, VKM F-5021, and VKM F-5022. To check the possible effects of the isolated strains on the plants, the isolates were used to infect sterile seedlings of Sarepta mustard (*Brassica juncea* L.). This model infection led to a moderate suppression of the photosynthetic apparatus activity and plant growth. Here, we present characteristics of the *F. commune* isolates: description of the conidial morphology, pigmentation, and composition of the mycelium fatty acids. Overall, this is the first description of the *Fusarium* cultures isolated from truffle fruiting bodies. Possible symbiosis of the *F. commune* strains with truffles and their involvement in the cooperative fatty acid production are proposed.

## 1. Introduction

The genus *Fusarium* was described in the 19th century: its study is related to the importance of these fungi for agriculture. Numerous *Fusarium* species are known as phytopathogens and etiological agents of plant diseases, the control of which remains relevant at present [1,2,3]. At the same time, certain species/strains of *Fusarium* can be beneficial for plant growth, in particular by suppressing more dangerous pests [4,5]. In general, we can conclude that these fungi are able to easily penetrate into and interact with plants [6,7]. The diversity of *Fusarium* is extremely large, and information about new species has been constantly updated; more than 50 new species were described just in 2022–2023 (https://www.mycobank.org (accessed on 14 May 2024)).

Until the present time, the search for new phytopathogenic/endophytic representatives of this genus has been mainly carried out by isolating strains from higher plants (https://www.mycobank.org (accessed on 14 May 2024)). Herewith, it has been shown that *Fusarium* fungi can grow together with other fungi: in the described cases, they act as pathogens for the fruiting bodies of those fungi [8,9]. Moreover, there are published denaturing gradient gel electrophoresis and metabarcoding data on the presence of this genus in the microbiome of truffle fruiting bodies and also in the surrounding soil [10,11]. It is already well known that the fruiting bodies of truffles contain a large number of various accompanying bacteria and fungi, which are involved in the formation of the total aromas, antioxidants, and fatty acids (FA) of commercial truffle mushrooms and can determine their composition [12,13,14,15].

Truffles themselves are rhizosphere ascomycete fungi and are capable of forming symbiosis with roots of higher plants [16]. The truffle microbiota can contain various, including endophytic, fungi [10,11,12,13,14,15]. Our objective was searching for accompanying fungal species, which can be isolated on usual nutrient media. We did not know in advance which species would predominate when isolated on certain media. This article presents the results of our study of the dominant *Fusarium* genus representatives isolated from the fruiting bodies of three truffle species; namely, *Tuber magnatum*, *T. melanosporum*, and *Choiromyces venosus*. The isolates were identified using molecular methods, mycelium morphology and pigments. To understand if the isolated strains take any part in the well-known activity of truffles, namely, their interaction with plants and production of antioxidants, we carried out separate experiments. The phytotoxicity of the isolated *Fusarium* strains against sterile mustard seedlings (*Brassica juncea* L.) was tested. We also estimated the FA content and composition of the mycelia and compared these values with those of the truffle fruiting bodies. This research is the first isolation of *Fusarium* strains from truffles that enables discussing a possible role of *Fusarium* in these associations.

## 2. Materials and Methods

### 2.1. Isolation of the Dominant Accompanying Mycelial Strains from Truffles

The fruiting bodies of the Piedmont white truffle (*T. magnatum*) and the Périgord black truffle (*T. melanosporum*) were obtained from Anna Kudryavtseva (Moscow, Russia), who purchased them as commercial products from different companies and at different times (Figure S1). The fruiting bodies of the Troitsky truffle *C. venosus* were collected in the forests of the Moscow region and provided for our research by Mikhail Generalov (Moscow, Russia) and A. Kudryavtseva (Moscow, Russia) (Figure S1). These species were chosen for our research and comparison because the genera *Tuber* and *Choiromyces* belong to the same truffle family, *Tuberaceae,* and have been shown to have common sets of genes [17]. The truffle specimens were stored at low temperature in accordance with the existing recommendations [18,19,20]. As stated above, the aim of the work was not to investigate the quality of commercial truffle fruiting bodies, but to identify the dominant species of possible associated fungi. In this regard, one sample of each type of truffle was used, with special attention to its safety and the further sterility of the isolation procedure.

Isolation of pure cultures of the dominating accompanying mycelial fungi from the truffle fruiting bodies was carried out under aseptic conditions. The surfaces of the fruiting bodies were burned over a burner flame, placed for 30 s in 80% ethanol, then for 30 s in a 0.5% NaOCl solution, washed with sterile water and dried with sterile filter paper [21,22]. Then, the outer layers of the truffle were sequentially cut off with sterile scalpels, and fragments of the inner gleba of the fruiting body (1 × 1 cm^2^) were cut out and transferred to Petri dishes (90 mm) on the surface of the nutrient media.

To isolate the mycelial strains and check any absence of bacterial contamination, the following media were used: malt agar (IBPM RAS, Pushchino, Russia), agar for counting heterotrophic bacteria (PCA) (plate count agar, Difco, MA, USA), dextrose peptone agar with a pH indicator (DPA) (dextrose casein peptone agar, Merck, Darmstadt, Germany). After a number of subcultures, the absence of bacterial contaminants was checked on a PCA medium. The homogeneity of the cultures was judged by the morphology of their colonies on various media, as well as by the morphology and organization of the mycelium under microscopy.

When isolating accompanying fungi from the truffle fruiting bodies, the goal was to isolate any dominant strains. Initially the authors assumed various accompanying species to be discovered in the fruiting bodies of different truffles. The nutrient media were not specific but classical, and selection by growth rate was expected to result in a wide range of isolates. In reality, just a few various types of colonies were found, and in each case only one predominantly grew on malt agar. Domination of *Fusarium* fungi in all truffles was an interesting surprise.

### 2.2. Identification of the Isolated Fungi by Morphology and Molecular Methods

Preliminary identification of the isolated strains was carried out by mycelial morphology after 7 and 14 days of their growth on a potato glucose agar (PGA, IBPM RAS, Russia) and synthetic nutrient-poor agar (SNA) [23].

DNA was isolated from the biomass of the week-old fungi using glass beads (300–500 µm in diameter) and a lysis buffer (Tris Base 50 mM, NaCl 250 mM, EDTA 50 mM, SDS 0.3%, pH 8) to destroy the cells. To amplify the ITS region of rDNA, primers ITS1f (5′-CTT GGT CAT TTA GAG GAA GTA) and LR5 (5′-TCC TGA GGG AAA CTT CG) were used. Amplification of the nucleotide sequence of the *TEF1a* gene was carried out using primers EF-983f (5′-GCY CCY GGH CAY CGT GAY TTY AT) and EF-2218r (5′-ATG ACA CCR ACR GCR ACR GTY TG). Primers ITS5 (5′-GGA AGT AAA AGT CGT AAC AAG G) and EF-983f were used to sequence the amplified DNA fragments. DNA sequencing was performed using the BigDye Terminator V3.1 Cycle Sequencing Kit (Applied Biosystems, Waltham, MA, USA), followed by the analysis of the reaction products on a 3130xl Genetic Analyzer sequencer (Applied Biosystems, USA).

To compare the nucleotide sequences of the ITS regions of rDNA and the *TEF1a* gene obtained after sequencing, we used data from the NCBI gene bank (https://www.ncbi.nlm.nih.gov/ (accessed on 14 May 2024)) and the MycoID database (https://www.mycobank.org/ (accessed on 14 May 2024)).

The obtained sequences were deposited in the GenBank database under accession numbers PP345623-PP345625 (ITS fragment) and PP333391-PP333393 (*TEF1a* fragment).

### 2.3. Microscopy

Phase contrast microscopy of the grown mycelia was performed using a Nikon Eclipse Ci microscope (Nikon, Tokyo, Japan) with a Jenoptik ProgResSpeedXTcore5 camera (Jenoptik, Jena, Germany).

Microscopic examinations of the plant roots colonized with the mycelial cultures and in the sterile blank experiments were carried out using a Zeiss Axio Imager A1 fluorescent microscope (Zeiss, Oberkochen, Germany).

### 2.4. Infection of the Isolated Fungi in Plants

Sterile seedlings of the Sarepta mustard (*B. juncea* L., variety Russkaya) were obtained from the Pushchino Branch of the Institute of Bioorganic Chemistry (BIBCh RAS, Pushchino, Russia). Plants were grown in a climatic chamber at a temperature of 22–24 °C, 16 h daylight, at a light intensity of 190 µM/m^2^ s (lamp L36 W/765, BASIC Cool Daylight, 2500 Lm, 220–240 V) and relative air humidity 65%. An MS agar medium [24] containing the basic salts necessary for growth and 0.7% agar (Duchefa Biochemie, Haarlem, The Netherlands) was used as a model soil. Infection of the sterile seedlings with mycelium cultures was carried out on the 7th day of fungal growth.

### 2.5. Analysis of the Photosystem II State in Plants Infected with Mycelium

Measurements of variable chlorophyll (Chl) fluorescence enables analyzing the state of photosystem II (PSII) in the plants. The analyses were carried out using a MULTI-COLOR PAM fluorometer (Heinz Waltz GmbH, Effeltrich, Germany). Leaves of mustard seedlings (adapted to darkness for at least 30 min) were used for the measurements. The maximum quantum yield of PSII (*F*_v_/*F*_m_) was calculated according to the formula

(*F*_m_ − *F*_0_)/*F*_m_, (1)

where *F*_0_ is the background Chl fluorescence and *F*_m_ is the maximum Chl fluorescence induced by a saturating 500 msec flash (λ = 625 nm, 12,000 µM/m^2^-s) of darkness-adapted leaves.

The effective quantum yield of Chl fluorescence (*Y*(II)), and the quantum yields of regulated (*Y*(NPQ)) and unregulated (*Y*(NO)) non-photochemical quenching of Chl fluorescence were measured in the leaves adapted to light intensity of 212 µM/m^2^-s, λ = 625 nm. The calculations were carried out according to the formulas recommended for the fluorometer MULTI-COLOR PAM, namely:

*Y*(II) = (*F*_m_′ − *F*)/*F*_m_′ (2)

*Y*(NPQ) = *F F*/*F*_m_′ − *F F*/*F*_m_ (3)

*Y*(NO) = *F F*/*F*_m_, (4)

where *F*_m_′ is the fluorescence maximum induced by the saturating flash, and *F* is the stationary level of Chl fluorescence in leaves adapted to light. Thus, the sum of these indicators is equal to:

*Y*(II) + *Y*(NPQ) + *Y*(NO) = 1. (5)

The relative electron transport rate (ETR) was calculated according to the formula

ETR = PAR × ETR-Factor × Y(II),(6)

where PAR is photochemically active radiation; ETR-Factor is the average value for PSII in photon absorption in the visible spectrum (400–700 nm) taken to be 0.42 for leaves of numerous plants.

### 2.6. Analysis of Secondary Metabolites

In studies of the secondary metabolites, the fungi were subjected to submerged cultivation in 750 mL flasks with 150 mL of the classical Czapek liquid medium (IBPM RAS, Pushchino, Russia) on a shaker (220 rpm) at 23 ± 1 °C in the dark for 11 days. The medium was inoculated with plugs from 7 day old cultures under stationary conditions in a 2% malt extract at a temperature of 23 ± 1 °C. The metabolites were extracted from the culture liquid filtrate at pH 3 by threefold extraction with chloroform. The combined chloroform extracts were dried over Na_2_SO_4_ (anhydrous). The treated solutions were filtered and evaporated on a rotary evaporator. The extracts were analyzed with thin-layer chromatography (TLC) on silica gel plates (Silica gel F254, Merck, Darmstadt, Germany) in a chloroform-methanol-25% ammonia system (90:10:0.1), and the metabolites were isolated by preparative TLC. Estimated UV spectra of the compounds were recorded on a UV-160A spectrophotometer (Shimadzu, Kyoto, Japan). The mass spectra of the compounds were recorded on an LCQ Advantage MAX quadrupole mass spectrometer (Thermo Finnigan, Egelsbach, Germany), using a single-channel syringe pump for direct injection of a sample into the chamber for chemical ionization at atmospheric pressure. Detailed information about the structure of the compounds was obtained by analyzing MS/MS spectra at a collision energy of 20–40%.

### 2.7. Cultivation for FA Analysis

It has been previously shown that solid-state fermentation (SSF) of *Fusarium* mycelium [25,26] is no less effective than submerged cultivation. SSF simplified biomass sampling for analyses without medium admixture. SSF for FA analysis was carried out on a simplified casein Hansen-Nielsen medium [27] at 24 °C for 2 weeks.

### 2.8. Fatty Acid Analysis

The fatty acid content and composition were analyzed both in the fruiting bodies of truffles, which served as mycelium isolation sources, and in the grown mycelia of the isolated strains. In all cases, the biomass was crushed using ultrasonic disintegration with cooling to avoid heating the biomass being destroyed, then the crushed samples were lyophilized using an Iney-4 installation (IBP RAS, Pushchino, Russia). Samples after lyophilization were methylated and the target FA were analyzed by the standard methods using an Agilent 5977B mass spectrometer (Agilent Technologies, Santa Clara, CA, USA) with a 7890B gas chromatograph and software (https://www.agilent.com/en/product/gas-chromatography/gc-systems/7890b-gc-system, accessed on 14 May 2024). An HP-5MS column (5% phenylmethylsilicone, 30 m × 0.25 mm × 0.25 μm) was used in the process [28,29]. The following analyses were carried out: (1) the FA C_6_–C_18_ were identified; (2) their ratio was determined (moles, %); (3) the FA weight content per unit of dry biomass was calculated. The reference acids for quantitative analysis were C_18:1ω9_, linoleic C_18:2ω9.12_, and gamma-linolenic C_18:3ω6,9,12_ (Sigma-Aldrich, St. Louis, MO, USA).

### 2.9. Statistical Analysis

Experimental analyzes were performed in triplicate. Data are presented as arithmetic means. The confidence intervals did not exceed ±5%. Statistical analysis was performed using the standard methods provided by Excel (https://support.microsoft.com/en-us/office/use-the-analysis-toolpak-to-perform-complex-data-analysis-6c67ccf0-f4a9-487c-8dec-bdb5a2cefab6, accessed on 14 May 2024). Analysis of variance (ANOVA) was used to determine the significance of the presented data. All data presented in the article are significant (*p* > 99%).

## 3. Results and Discussion

### 3.1. Morphological Identification of Isolated Strains

Strains of the most abundant filamentous fungi, hereinafter referred to as strains BT2, ChT, and TT, were isolated from the Piedmont white, Périgord black, and Troitsky truffles, respectively.

**Figure 1:**
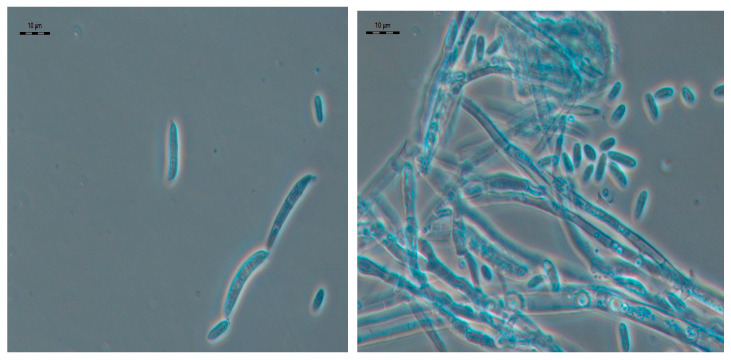
Micromorphology of strain BT2 as a typical feature of the isolates (grown on the SNA medium, 10 days). Scale bar, 10 µm.

Microscopy of the fungal cultures revealed macro- and microconidia in the preparations, typical of and common to the genus *Fusarium* Link (Figure 1). Macroconidia were found to form in aerial mycelium. The macroconidia were awl-shaped, slightly sickle-shaped, elliptically curved or almost straight, with an equal diameter over most of the length, with a thin shell, gradually tapering towards both ends, with a gradually tapering non-elongated upper cell, narrowed towards the base, with a clearly defined stalk or papilla, usually with 3 septa, 25–50 × 3–5 µm in size. Microconidia were ovoid, oval, unicellular, formed in chains or in false heads, from simple phialides. The size of unicellular microconidia was 4–8 × 1.5–3.5 µm, with 1 septum 9–30 × 2–5 µm. Chlamydospores were abundant, apical and intermediate, hyaline, 8–12 µm in diameter.

It should be noted that the similarity of micromorphological characteristics of the three isolates allowed us to classify them according to their morphology as belonging to *F. oxysporum* (Schlecht.) Snyd. et Hans [30]. At the same time, the species *F. oxysporum* is morphologically similar to *F. commune*. Both of these species are characterized by the formation of conidia on aerial mycelium on short monophialides and the abundant formation of chlamydospores, terminal and in chains. The only morphological difference was the formation of long monophialides in *F. commune* in addition to numerous polyphyalides [31].

**Figure 2:**
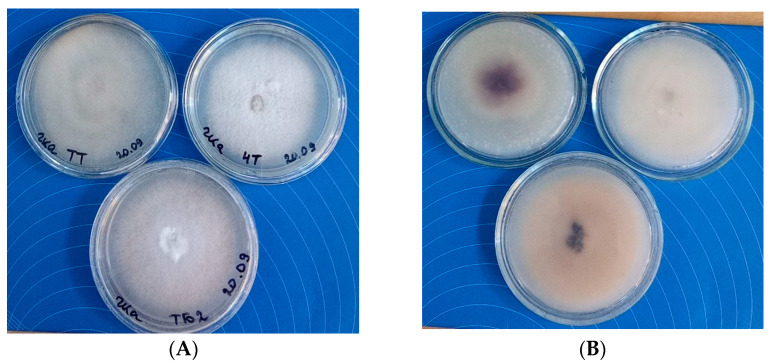
Colonies of TT, ChT, and BT2 strains on PGA in the Petri dishes, diameter 90 mm, 7 days. (**A**) Top view; (**B**) reverse view.

Figure 2 shows colonies of all three *Fusarium* isolates grown on potato glucose agar (PGA). All strains were fast growing, the aerial mycelium was filmy-cobwebby, low, white at the beginning of growth; the reverse was fawn, but with age it turned pinkish-carmine-lilac. It should be noted that with age, the isolates differed in the color of aerial mycelium. On PGA, the aerial mycelium of BT2 turned pink, while the reverse acquired a pinkish-yellow color, and the center of the colony turned black and inky. When grown on malt agar, BT2 was the only one that released black pigment into the medium. In the ChT isolate, the color of the mycelium and of the reverse practically did not change with age, but in the TT isolate it acquired a grayish tint, and the reverse in the center of the colony turned pale brown.

### 3.2. Molecular Identification of Fusarium Isolates

The obtained nucleotide sequences of the ITS regions in the rDNA of strains BT2 and ChT were identical; the sequence for strain TT differed from them by four variable nucleotides. Use of the NCBI genebank data and the MycoID database showed the presence of conspecific strains (99.5–100% similarity), isolated primarily as root endophytes of various plants from America and Asia (Table 1). However, unambiguous species identification of the isolated strains using these data is difficult, since all reference strains must be used for comparison.

According to genetic data, the closest species of the genus *Fusarium* to the obtained isolates were *F. acutatum* CBS 402.97T (similarity 99.42%), *F. concentricum* CBS 450.97T (99.42%), *F. dlaminii* CBS 738.97T (99.25%), and *F. annulatum* CBS 258.54T (99.24%). Currently, not all members of the genus *Fusarium* are genetically characterized by their ITS region of rDNA [32]. For more accurate species identification, we carried out an analysis of the nucleotide sequence of the *TEF1a* gene. Based on the data obtained, strains BT2, TT, and ChT were classified as the species *F. commune* Skovgaard, O’Donnel et Nirenberg. The similarity of *TEF1a* gene nucleotide sequences of the isolates to *F. commune* strain NRRL 28387 (KU171720) was 99.59%.

In connection with the identification carried out here, all three isolated cultures were designated as *F. commune* with some differences at the strain level. These three strains were deposited in the All-Russian Collections of Microorganisms (VKM) with the following collection designations: BT2 = *F. commune* VKM F-5020, ChT = *F. commune* VKM F-5021, TT = *F. commune* VKM F-5022.

### 3.3. Effect of Fusarium Infection on Mustard Seedlings

**Figure 3:**
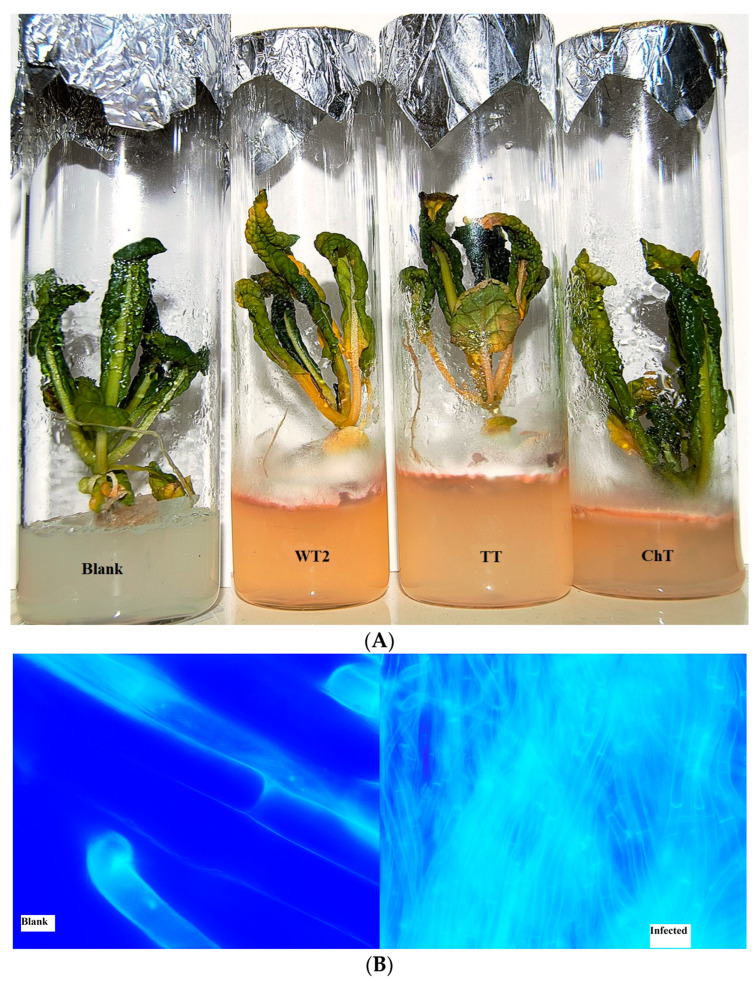
(**A**) General view of mustard seedlings (*B. juncea*) infected with *F. commune* strains BT2, TT, and ChT versus the sterile seedling (control). Soil was replaced with an MS agar medium. Growth time, 7 days after infection. (**B**) View of the control and infected roots 7 days after infection (light fluorescent microscopy); white scale bar with the designations “blank” and infected” is 10 µm.

Due to the fact that numerous *Fusarium* species and strains, including *F. commune*, show endophytic and/or phytopathogenic behavior, we tested our isolated strains in experiments with mustard seedlings (*B. juncea*). Within 7 days of infection, according to direct visual observations, the MS agar medium changed its color compared to the control due to the released pigments, while leaves of the infected plants turned yellow (Figure 3A). Microscopy of the roots showed that the mycelium abundantly overgrew the roots (Figure 3B). It should be mentioned that a noticeable deterioration in growth also could be associated with an indirect effect of fungi as competitors for nutrients in the environment. Thus, the infection effect was evaluated not only by the general state of plants but also by the measured response of their PSII as a sensitive indicator of the photosynthetic apparatus state.

**Figure 4:**
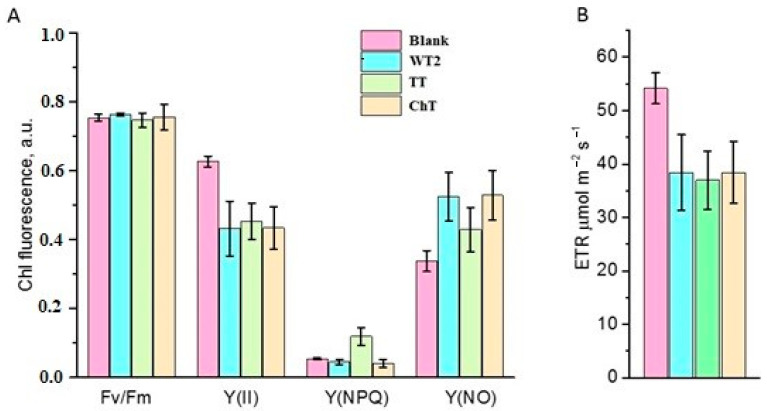
PAM-parameters related to PSII states obtained for control and infected plants. (**A**) Values of the maximum (*F*v/*F*m) and effective (*Y*(II)) quantum yields of Chl fluorescence of PSII; values of the quantum yield of regulated (*Y*(NPQ)) and unregulated (*Y*(NO) non-photochemical quenching of Chl fluorescence of PSII in the control and infected plants. (**B**) Relative electron transport rate (ETR) through PSII in the control and infected plants.

The maximum quantum yield of the Chl fluorescence (*F*_V_/*F*_m_) reflecting the maximum potential of the PSII for photochemical charge separation was the same in the sterile (control) and infected plants (Figure 4A). In all cases, the *F*_V_/*F*_m_ ratio was approximately 0.75, which is very close to the typical value for the vast majority of higher plants [33]. Thus, inoculation with *F. commune* strains did not lead to critical damage for the PSII reaction centers.

Adaptation of plants to light usually leads to a decrease in the quantum yield of Chl fluorescence, denoted as an effective quantum yield (*Y*(II)). In the case of non-inoculated (control) plants, the *Y*(II) value was lower than *F*_V_/*F*_m_ by only 17%, while for infected plants this value decreased more drastically to 39–43% of the maximum quantum yield (Figure 4A). The measured value of *Y*(II) in infected plants was 28–31% lower than in the control plants; these results are in good agreement with the data on the relative electron transport rate (ETR) through the PSII (Figure 4B). In the case of infected plants, the ETR value was 30–31% lower than that of the controls.

Non-photochemical quenching of Chl fluorescence is related to various mechanisms of absorbed light energy dissipation [34]. It can be carried out by unregulated mechanisms (*Y*(NO)) including photodamage to PSII centers, disruption of the association of the antenna complexes with PSII, etc. In the conducted experiments, the proportion of *Y*(NO) in plants infected with *F. commune* was obviously higher (Figure 4A). However, while in plants infected with BT2 and ChT strains the value of *Y*(NO) was 55–56% higher in comparison with the control plants, in the case of infection with strain TT the increase was ~27%.

At the same time, the contribution of the regulated mechanisms of non-photochemical quenching (*Y*(NPQ)) of Chl fluorescence (the xanthophyll cycle, PsbS protein dimerization, etc. [34]) significantly increased in plants only infected with the TT strain. It amounted to 120% of the value for the control plants and those infected with BT2 and ChT strains. One of the possible explanations for the detected differences may be the indirect effect of infection with the TT strain on the acidification of the thylakoid lumen, which is the main trigger of the mechanisms of regulated non-photochemical quenching of Chl fluorescence [34]. Thus, all three investigated strains demonstrated an ability of endophytic growth and a slight phytopathogenic effect.

### 3.4. Pigments of Isolated F. commune Strains

**Figure 5:**
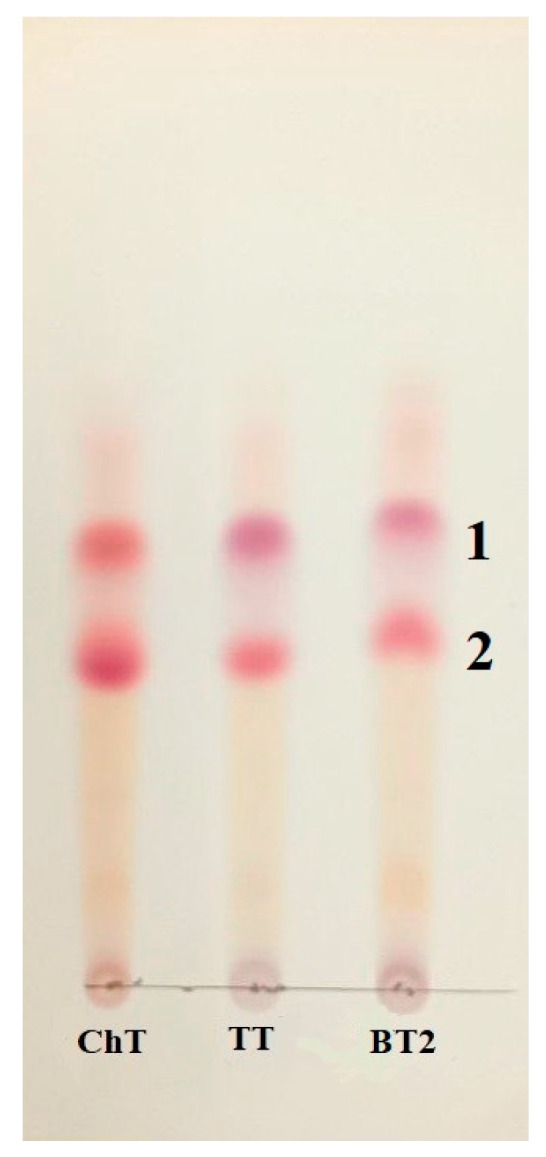
Pigments isolated from the *F. commune* extracts with TLC. Designations of the strains and numbers of isolated pigments are shown in the figure.

The Czapek liquid medium was extracted after the growth of *F. commune* strains, and the extracted set of metabolites was identical for all three isolates, consisting mainly of two red-burgundy pigments (Figure 5). Metabolite 1 had a UV spectrum with absorption bands at *λ*_max_ of 222, 266, 343, and 493 nm. The mass spectrum of metabolite **1** showed a positive molecular ion 319 (M + H)^+^ and a negative molecular ion 317 (M − H)^−^. Metabolite 2 had the *λ*_max_ at 222, 262, 292, 350 (sh.) 498, and 531 nm. The molecular ions had masses of 307 (M + H)^+^ and 305 (M − H)^−^. According to the analytical methods, the data indicated the secretion of polyketide pigments of naphthaquinone nature. Metabolite 1 was fusarubin, while metabolite 2 was not identified.

The ability to produce naphthoquinones is widespread among fungi, especially among representatives of the genus *Fusarium* [35,36]. The genus *Fusarium* is collectively capable of producing three polyketide pigments: aurofusarin, bicaverine, and fusarubins (including bostricoidin, javanicin) [37]. The greatest number of the known *Fusarium* species use aurofusarin and bikaverin for mycelium pigmentation and fusarubins for perithecia pigmentation. These metabolites have known antimicrobial, antitumor, and phytotoxic activities [38,39,40]. Fusarubin has shown some cytotoxicity against human leukemia cells and also inhibits proliferation and increases apoptosis in cell lines derived from hematological cancer cells [41,42]. It is also active against hazardous bacteria such as *Staphylococcus aureus*, *Escherichia coli*, *Pseudomonas aeruginosa,* and *Bacillus megaterium*. The phytotoxic effect of naphthoquinones can be associated with a decrease in the level of NAD(P)H in plant cells and the formation of superoxide radicals [43].

### 3.5. Fatty Acids in F. commune Mycelia

Preliminary experiments with cultivating strains on various nutrient media allowed us to choose a simplified casein Hansen-Nielsen medium as the preferred one. Table 2 shows the FA content in the mycelia of *F. commune* strains VKM F-5020 (BT2), VKM F-5021 (ChT), and VKM F-5022 (TT). All three strains showed a similarity in composition of the dominating FA. The highest concentration was shown for unsaturated linoleic acid (C_18:2ω9,12_). The high production of unsaturated fatty acids by *F. commune* stimulates a discussion on their possible use in biotechnology.

### 3.6. Discussion Regarding the Symbiosis of Truffles and F. commune

The single dominating species of the genus *Fusarium*, namely *F. commune*, was isolated from three various species of truffle fruiting bodies. This coincidence does not seem to be accidental and may indicate a symbiosis of these rhizosphere ascomycete fungi. The ability of endophytic *Fusarium* to attach to the roots of higher plants should also help the truffles to grow on the roots.

Of additional interest is the coincidence of the FA composition found in the fruiting bodies of truffles in [44,45,46] with our data for mycelia of the isolated *Fusarium* strains. We also analyzed the FA in the fruiting bodies of truffles, which served as the sources for our isolation. Quantitative analysis of the FA composition in the fruiting bodies of *T. magnatum*, *T. melanosporum*, and *C. venosus* discovered unsaturated acid with predominant linoleic acid (Table 3); its share of the total measured FA content ranged from 52% to 64% of the measured C_6_–C_18_ sum. Basically, the fatty acid profiles of the analyzed truffle samples were similar. Recalculation of the FA content per dry biomass of the truffle fruiting body showed that their concentration ranged from 5.51% in *T. magnatum* to 8.14% in *T. melanosporum* (Table 4). On the whole, the qualitative and quantitative compositions of fatty acids in our truffle specimens were close to the known data.

In turn, a similarity of FA compositions in *F. commune* mycelium and in the truffles suggests some involvement of *Fusarium* in FA production in the fruiting bodies. Possibly, production of useful substances in truffle fruiting bodies is ensured by the symbiotic microbiome as a whole. High production of valuable unsaturated fatty acids by the isolated *Fusarium* strains (Table 4) enables considering them as commercial producers; however, they can be used only under safety conditions for endophytic fungi.

It should be mentioned that the detected moderate phytotoxicity of *F. commune* strains found in the fruiting bodies of truffles does not cast doubt on the food suitability of these truffles. The reasons for these conclusions are the following. The phytotoxicity of *F. commune* when growing on plants is not high enough to harm the human gastrointestinal tract during digestion. The amount of *F. commune* detected (just a few germinations from 2–5-g samples) suggests its low percentage in the truffle. The isolated *F. commune* cultures did not produce any mycotoxins, either.

## 4. Conclusions

This work presents the first report on the isolation of *Fusarium* from truffle fruiting bodies into pure cultures. All isolates were identified by molecular tools as *F. commune*, with some differences between strains in their morphology and pigmentation. The taxonomic coincidence of isolates is evidence of *Fusarium’s* common presence in truffle microbiomes. At the same time, the detected differences between the isolated strains may indicate some additional specificity of *F. commune* strains or subspecies to different truffle species/genus.

Infection of mustard seedlings with *Fusarium* cultures confirmed the endophytic and moderately phytotoxic properties of the isolates due to the suppression of photosynthetic activity. The detected phytotoxicity can be partially explained by the action of the discovered naphthoquinone pigments, including fusarubin, which was previously shown to have a variety of biological, including phytotoxic, activities.

In turn, the endophytic properties of *F. commune* isolates can play a significant role in their symbiosis with truffles and contribute to the establishment of the truffle microbiome on the roots of higher plants. In addition, the coincidence of the FA composition in the *Fusarium* mycelium and in the truffle fruiting bodies can also serve as an indirect confirmation of the symbiotic production of FA by ascomycetes in the truffle fruiting bodies.

## Figures and Tables

**Table 1:** Information on conspecific strains of the genus *Fusarium* according to their ITS regions of rDNA.

Strains	Source of Isolation	Region of Isolation	NCBI Designation
Fungal endophyte ARIZ: DM0010	*Persicaria amphibia*, roots	USA	KF673560
*F. oxysporum* f. sp. *raphani* (*F. commune*) MAFF 240304	*Brassica campestris* var. *glabra*, roots	Japan	AB586993
*F. oxysporum* f. sp. *rapae* (*F. commune*) MAFF 240321	*B. campestris* var. *perviridis*, stalk	Japan	AB586994
*Fusarium* sp. FEB10C	*Bromus tectorum*, leaves	USA	KT347157
*Fusarium* sp. GLBRC403	*Panicum virgatum*, roots	USA	OM106600
*Fusarium* sp. GLBRC351	*P. virgatum*, roots	USA	OM106548
*Fusarium* sp. GLBRC336	*P. virgatum*, roots	USA	OM106534
*Fusarium* sp. 880633-31-06	*Musa itinerans*, seeds	Vietnam	MW299357

**Table 2:** FA content in *F. commune* mycelia grown on a Hansen medium for 2 weeks, %. The FA concentrations over 10% are highlighted in bold. Confidence intervals did not exceed ±5%.

Fatty Acids (FA)	FA Concentration, %		
	VKM F-5020 (BT2)	VKM F-5021 (ChT)	VKM F-5022 (TT)
C_6:0_	0.42	0.00	0.00
C_8:0_	0.35	0.10	0.00
C_10:0_	2.39	0.10	0.87
C_12:0_	0.00	1.35	0.78
C_14:1_ω_9_	0.00	0.00	0.00
C_14:0_	1.73	1.25	3.02
iC_15:0_	0.00	0.00	0.00
aC_15:0_	0.00	0.00	0.00
iC_16:0_	0.00	0.00	0.00
C_16:1_ω_9_	0.00	0.00	1.19
C_16:0_	**31.48**	**32.46**	**16.56**
aC_17:0_	0.00	0.00	0.00
C_18:3_ω_6,9,12_	0.00	0.00	**19.30**
C_18:2_ω_9,12_	**47.29**	**43.99**	**34.15**
C_18:1_ω_9_	**10.58**	**15.47**	**18.95**
C_18:0_	5.63	5.28	5.18
Other	0.13	0.00	0.00

**Table 3:** FA content of the truffle fruiting bodies, %. The FA content exceeding 10% is highlighted in bold. Confidence intervals did not exceed ±5%.

Fatty Acids (FA)	FA Content, %		
	*T. magnatum*	*T. melanosporum*	*C. venosus*
C_6:0_	0.00	0.00	0.00
C_8:0_	0.00	0.00	0.00
C_10:0_	0.00	0.00	0.00
C_12:0_	0.00	0.00	0.00
C_14:1_ω_9_	0.00	0.00	0.00
C_14:0_	0.00	0.00	0.00
iC_15:0_	0.00	1.04	0.00
aC_15:0_	0.00	3.27	0.00
iC_16:0_	0.00	2.46	0.00
C_16:1_ω_9_	0.00	1.04	0.58
C_16:0_	**16.38**	**11.24**	**17.71**
aC_17:0_	0.00	1.54	0.00
C_18:3_ω_6,9,12_	0.00	0.00	0.00
C_18:2_ω_9,12_	**53.43**	**54.95**	**62.45**
C_18:1_ω_9_	**26.90**	**20.95**	**15.62**
C_18:0_	3.28	2.79	3.64
Other	0.00	0.72	0.00

**Table 4:** Total FA content in the truffle fruiting bodies and *F. commune* mycelium, % of dry biomass. Confidence intervals did not exceed ±5%.

FA Content, % of Dry Biomass					
*T. magnatum*	*T. melanosporum*	*C. venosus*	*F. commune* VKM F-5020 (BT2)	*F. commune* VKM F-5021 (ChT)	*F. commune* VKM F-5022 (TT)
5.51	8.14	7.04	21.76	9.98	31.68

## Data Availability

The original contributions presented in the study are included in the article/supplementary material, further inquiries can be directed to the corresponding authors.

## Supplementary Materials

The following supporting information can be downloaded at: https://www.mdpi.com/article/10.3390/jof10070463/s1, Figure S1: General view and sections of the truffle fruiting bodies: A—*T. magnatum*, B—*T. melanosporum*, C—*C. venosus*.
