# Supplementary material for: Phytotoxic Strains of Fusarium commune Isolated from Truffles
Source: J Fungi (Basel). 2024 Jun 29;10(7):463. doi: 10.3390/jof10070463 (PMC11278203; doi:10.3390/jof10070463)
Supplement: Supplementary file 1 [file jof-10-00463-s001.zip › jof-3037763-supplementary.pdf]

Supporting Information
for

Phytotoxic Strains of *Fusarium commune* Isolated from Truffles

Anton Zvonarev [1], Vasily Terentyev [2], Valentina Zhelifonova [1], Tatiana Antipova [1,3,*], Boris Baskunov [1], Aleksander Avtukh [1], Tatiana Abashina [1], Aleksey Kachalkin [1,4], Mikhail Vainshtein [1,*] and Anna Kudryavtseva [5]

[1] G.K. Skryabin Institute of Biochemistry and Physiology of Microorganisms, Federal Research Center "Pushchino Scientific Center for Biological Research of the Russian Academy of Sciences", Pushchino 142290, Russia; zvonarevibpm@gmail.com (A.Z.); avtukh@rambler.ru (A.A.); kachalkin_a@mail.ru (A.K.)
[2] Institute of Basic Biological Problems, Federal Research Center "Pushchino Scientific Center for Biological Research of the Russian Academy of Sciences", Pushchino 142290, Russia
[3] All-Russian Institute of Plant Protection, Pushkin 196608, Russia
[4] The Faculty of Soil Science, M.V. Lomonosov Moscow State University, Moscow 119234, Russia
[5] Independent Researcher, Moscow 115533, Russia
* Correspondence: tatantip@pbcras.ru (T.A.); vain@pbcras.ru (M.V.); Tel.: +7-4967-732677 (M.V.)

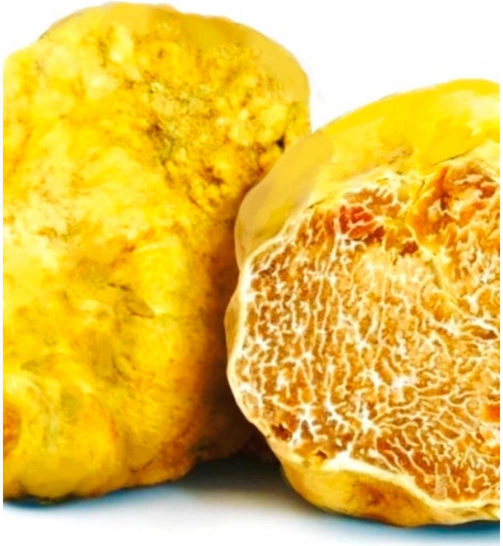

A

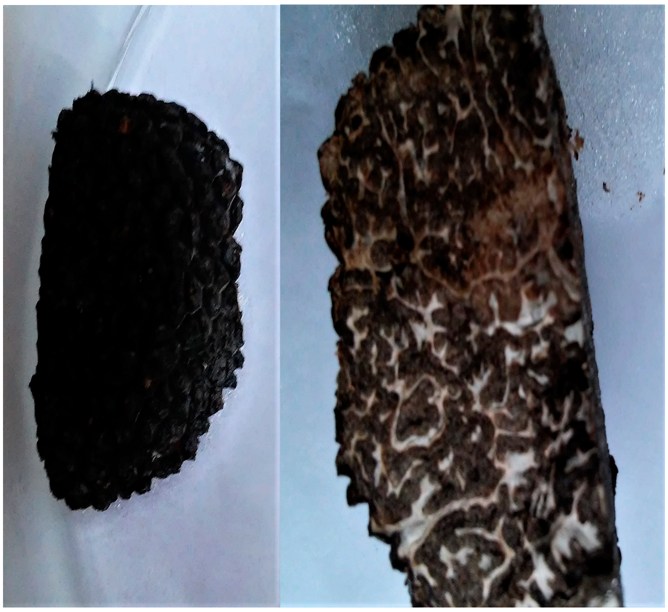

B

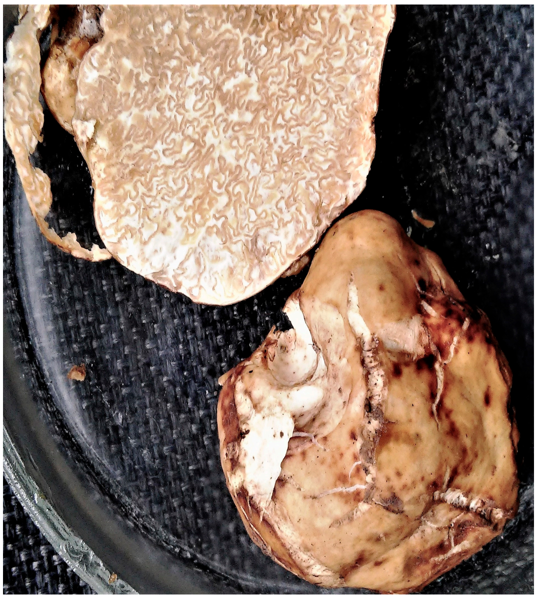

C

Figure S1 – General view and sections of the truffle fruiting bodies:

A - *T. magnatum,* B - *T. melanosporum,* C - *Ch. venosus*.
